# Supplementary material for: Global and local tension measurements in biomimetic skeletal muscle tissues reveals early mechanical homeostasis
Source: eLife. 2021 Jan 18;10:e60145. doi: 10.7554/eLife.60145 (PMC7906603; doi:10.7554/eLife.60145)
Supplement: Transparent reporting form [file elife-60145-transrepform.pdf]

eLIFE
1st Floor
24 Hills Road
Cambridge CB2 1JP, UK
P 01223 855340
W elifesciences.org
T @elife

eLife's transparent reporting form

We encourage authors to provide detailed information *within their submission* to facilitate the interpretation and replication of experiments. Authors can upload supporting documentation to indicate the use of appropriate reporting guidelines for health-related research (see EQUATOR Network), life science research (see the BioSharing Information Resource), or the ARRIVE guidelines for reporting work involving animal research. Where applicable, authors should refer to any relevant reporting standards documents in this form.

If you have any questions, please consult our Journal Policies and/or contact us: editorial@elifesciences.org.

Sample-size estimation

- You should state whether an appropriate sample size was computed when the study was being designed
- You should state the statistical method of sample size computation and any required assumptions
- If no explicit power analysis was used, you should describe how you decided what sample (replicate) size (number) to use

Please outline where this information can be found within the submission (e.g., sections or figure legends), or explain why this information doesn't apply to your submission:

Pilot experiments with low replicates already showed signifcances which were further confirmed during experiment repetitions. Every experiment was repeated at least at two independent days. Additionally, comparable published studies of biomimetic skeletal muscle tissue research used similar sample-sizes. Description of the sample-sizes for each experiment is outlined in the figure legends.

Replicates

- You should report how often each experiment was performed
- You should include a definition of biological versus technical replication
- The data obtained should be provided and sufficient information should be provided to indicate the number of independent biological and/or technical replicates
- If you encountered any outliers, you should describe how these were handled
- Criteria for exclusion/inclusion of data should be clearly stated
- High-throughput sequence data should be uploaded before submission, with a private link for reviewers provided (these are available from both GEO and ArrayExpress)

Please outline where this information can be found within the submission (e.g., sections or figure legends), or explain why this information doesn't apply to your submission:

Biological replicates (N) are defined as the number of biomimetic muscle tissues that were raised and used. Technical replicates (n) are defined as the number of elastic PAA beads that were analysed and in Figure 4E 'n' is defined as the number of myotubes that were measured. These definitions are clearly stated in the section "Methods and Materials" in subsection "Statistical analysis".
In every figure legend 'N' and 'n' is outlined for all the subpanels of the quantified data.
Data were excluded if a raised tissue was not showing contractile responses or did not show characteristic morphological myotube formation ahead of the scheduled experimental timepoint.

Statistical reporting

- Statistical analysis methods should be described and justified
- Raw data should be presented in figures whenever informative to do so (typically when N per group is less than 10)
- For each experiment, you should identify the statistical tests used, exact values of N, definitions of center, methods of multiple test correction, and dispersion and precision measures (e.g., mean, median, SD, SEM, confidence intervals; and, for the major substantive results, a measure of effect size (e.g., Pearson's r, Cohen's d)
- Report exact p-values wherever possible alongside the summary statistics and 95% confidence intervals. These should be reported for all key questions and not only when the p-value is less than 0.05.

Please outline where this information can be found within the submission (e.g., sections or figure legends), or explain why this information doesn't apply to your submission:

Under section "Methods and Materials" in subsection "Statistical analysis" it is described that all data are shown throughout the whole manuscript as mean ±SD if not mentioned differently and that all significances were calculated using an unpaired t-test since the analysed data are normal distributed. In the subsection "Statistical analysis" also the levels of significances are clearly stated.
Raw data points for each technical replicate is indicated on all bar graphs.

(For large datasets, or papers with a very large number of statistical tests, you may upload a single table file with tests, Ns, etc., with reference to sections in the manuscript.)

Group allocation

- Indicate how samples were allocated into experimental groups (in the case of clinical studies, please specify allocation to treatment method); if randomization was used, please also state if restricted randomization was applied
- Indicate if masking was used during group allocation, data collection and/or data analysis

Please outline where this information can be found within the submission (e.g., sections or figure legends), or explain why this information doesn't apply to your submission:

Masking and randomization was not used during this study.

Additional data files ("source data")

- We encourage you to upload relevant additional data files, such as numerical data that are represented as a graph in a figure, or as a summary table
- Where provided, these should be in the most useful format, and they can be uploaded as "Source data" files linked to a main figure or table
- Include model definition files including the full list of parameters used
- Include code used for data analysis (e.g., R, MatLab)
- Avoid stating that data files are "available upon request"

Please indicate the figures or tables for which source data files have been provided:

eLife Sciences Publications, Ltd is a limited liability non-profit non-stock corporation incorporated in the State of Delaware, USA, with company number 5030732, and is registered in the UK with company number FC030576 and branch number BR015634 at the address 1st Floor, 24 Hills Road, Cambridge CB2 1JP | August 2014

All data supporting the findings of this study are available within the article and supplements. Programs and analysis code can be found on GitHub as mentioned in the main text. Source data of all graphs were uploaded in the Manuscript Items as excel or matlab files.
